# Evaluating the Effectiveness of Food Safety Policies in Portugal: A Stakeholder-Based Analysis of Challenges and Opportunities for Food Safety Governance

**DOI:** 10.3390/foods14091534

**Published:** 2025-04-27

**Authors:** Júlia Rodrigues, Cristina Saraiva, Juan García-Díez, José Castro, Alexandra Esteves

**Affiliations:** 1 Department of Veterinary Sciences, School of Agricultural and Veterinary Sciences, Universidade de Trás-os-Montes e Alto Douro, Quinta de Prados, 5000-801 Vila Real, Portugal; julia.rodrigues2011@gmail.com (J.R.); crisarai@utad.pt (C.S.); alexe@utad.pt (A.E.); 2 Veterinary and Animal Research Centre (CECAV), University of Trás-os-Montes e Alto Douro, Quinta de Prados, 5000-801 Vila Real, Portugal; 3 Associate Laboratory for Animal and Veterinary Science (AL4AnimalS), 1300-477 Lisboa, Portugal; 4 Department of Natural Resources and Environment, College of Agriculture, Polytechnic of Bragança, Campus Sta. Apolonia, 5300-253 Bragança, Portugal; mzecast@ipb.pt

**Keywords:** food safety, food policy, public health, public politics, stakeholders

## Abstract

Food safety is a fundamental component of public health, economic stability, and consumer confidence. In Portugal, the National Integrated Multiannual Control Plan (NIMCP) serves as a strategic framework for ensuring food safety and compliance with European Union food regulations. However, challenges persist in policy implementation and enforcement, as well as in stakeholder engagement, which impact the effectiveness of food safety governance. This study employs a mixed-methods approach to assess stakeholder perceptions of the NIMCP, focusing on levels of compliance, barriers to its implementation, and potential improvement measures. A structured online survey was conducted with 217 stakeholders, including representatives of public institutions, private entities, associations, and consumer groups. The survey assessed the perceived importance of the NIMCP objectives and levels of compliance and identified barriers, such as a lack of communication between public entities, the dispersion of responsible agencies, and insufficient dissemination of information. The results indicate that stakeholders perceive a satisfactory level of compliance with the NIMCP objectives, especially in areas such as animal health and risk control. However, challenges persist in ensuring plant health and implementing official controls. Furthermore, stakeholders highlight systemic inefficiencies and resource constraints. The main barriers include fragmented governance structures, limited inter-agency collaboration, and insufficient professional training. Stakeholders proposed various improvement measures, emphasizing the need for better coordination, planning, and communication, including awareness campaigns for operators, the creation of an integrated IT network, and the development of training programs. The Analytical Hierarchy Process (AHP) revealed that risk control and consumer protection are top priorities for all stakeholder groups, while plant and animal health receive lower priority. The study concludes that while the NIMCP is generally perceived as effective, addressing systemic issues such as coordination, communication, and resource allocation is essential to improving food safety governance. Policymakers are encouraged to adopt a more structured and integrated approach to improve implementation of the NIMCP, ultimately strengthening public health protection and consumer confidence in the food supply chain.

## 1. Introduction

Food safety is a fundamental pillar of public health, economic stability, and consumer trust. Ensuring the safety of food products is essential to prevent foodborne illnesses, which globally affect an estimated 600 million people annually, leading to 420,000 deaths [1], over 140,000 in Europe [2]. Beyond health risks, food safety failures can have severe economic repercussions, including trade restrictions, market losses, and reputational damage for food industries and national governments [3]. Thus, to improve public health and guarantee the protection of consumers, the European Union (EU) has displayed several food safety regulations designed to maintain high hygiene standards, risk monitoring, and traceability across the food supply chain, from farm to fork [4,5,6,7,8,9]. Portugal, as an EU member, adheres to harmonized food safety policies under the European Food Safety Authority (EFSA) and the General Food Law [7]. However, despite regulatory alignment with EU directives, challenges persist in policy implementation, enforcement, and stakeholder engagement, impacting the overall effectiveness of food safety governance [10]. Fragmented oversight, inconsistent enforcement, and gaps in inter-agency coordination create barriers to compliance, particularly for private sector food operators and consumers who rely on transparent and effective regulatory systems.

In Portugal, food safety governance is managed through multiple regulatory bodies such as the national veterinary authority (DGAV) (responsible for food inspection, veterinary health, and agricultural safety), the food and economic safety authority (ASAE) (mainly aimed to control of food fraud, food labelling or hygiene inspections, mainly at the retail level), regional agricultural and veterinary offices (providing localized food safety oversight) or camaras municipais (i.e., townhalls) (aimed to food hygiene licensing and veterinary public affairs) among others. All official bodies aim to implement the National Integrated Multiannual Control Plan (NIMCP), a strategic plan designed to ensure food safety and quality, protect human, animal, and plant health, and ensure compliance with European and national regulations throughout the agri-food chain, from farm to fork [11]. Its implementation is carried out through an approach based on risk assessment, and it is continuously monitored and updated to address emerging challenges and risks related to food safety and public health.

The NIMCP’s ultimate objective is to ensure a high level of protection of human, animal and plant health, as well as animal welfare and the environment. It also aims to contribute to the proper functioning of the internal market by ensuring that products in circulation comply with established standards, promoting fair trading practices and safeguarding the interests of consumers by guaranteeing the safety and integrity of food and feed.

Its application complies with the rules established in [9] and acts in ten areas: food products (food safety, commercial practices and labelling), intentional release of genetically modified organisms (GMOs) into the environment, animal feed (feed safety), animal health (disease control and animal movements), animal by-products (prevention of risks to human and animal health), animal welfare (breeding, transport and slaughter conditions), protection of plant pests, plant protection products and pesticides (sustainable use), organic production and labelling of organic products, and use and labelling of products with Protected Designation of Origin, Protected Geographical Indication) and Traditional Specialty Guaranteed. The implementation of the NICMP affects all stakeholders in the food chain, from farmers to consumers. Since each stakeholder has its own interests; each of them frequently expresses concerns about the inefficiencies of the NIMCP regarding aspects such as interpretation and application of food policy, the overlapping of responsibilities, bureaucratic obstacles, and the high economic burden, both in terms of human resources and financial costs [12,13].

Although there is extensive research on food safety compliance along the food chain, studies regarding stakeholders’ perceptions about the effectiveness of public policies on food safety are scarce [14]. Thus, this study aims to answer the question of how stakeholders in Portugal perceive the implementation, barriers, and opportunities for improvement of the National Integrated Multiannual Control Plan (NIMCP) for food safety governance. In particular, it explores the challenges associated with the implementation of the NIMCP, as well as the improvement measures proposed by stakeholders to enhance its effectiveness, policy efficiency, and public confidence in food safety governance.

## 2. Materials and Methods

### 2.1. Study Design

This study employed a mixed-methods approach to evaluate stakeholder perceptions of food safety policies in Portugal, focusing on the National Integrated Multiannual Control Plan (NIMCP). A structured online survey was used to collect quantitative and qualitative data from key stakeholders across the food supply chain. The survey was designed to assess the perceived importance of NIMCP objectives, the level of compliance, and potential barriers to effective implementation. Additionally, scenario analysis was conducted to evaluate alternative policy approaches based on stakeholder influence in decision-making.

### 2.2. Participants and Sampling

The survey targeted representatives from public institutions, private entities, associations, and consumer groups involved in food safety governance, classified into five main stakeholder groups: associations of animal production, private sector animal producers, public animal production agencies, consumers and consumer associations, and public food safety control agencies. Participants were recruited through professional networks, food safety organizations, and direct invitations via email and industry forums.

All participants must be representatives of one of five pre-defined stakeholder groups: (i) animal production associations (e.g., federations, cooperatives), (ii) private sector animal producers (e.g., CEOs, livestock or food production companies), (iii) public agencies related to animal production (e.g., governmental institutions regulating food production), (iv) consumers and consumer associations (e.g., consumer rights organizations or individuals with an active interest in food safety policies), or (v) public food safety control agencies (e.g., regulatory or inspection bodies). Thus, all participants from governmental entities must have direct or indirect involvement in the management, control, and organization of food safety, participate in the implementation of food safety-related policies, or intervene in the enforcement of food legislation throughout the entire food chain in Portugal. For both private sector and consumer participants, these included those with decision-making or operational responsibilities related to food safety, as well as those with a special interest in food safety, respectively. Furthermore, all participants must be based in Portugal and actively participate in the national food safety system. Individuals or entities not directly involved in food safety governance, as well as participants who did not complete the survey or provided inconsistent or incomplete data, were excluded from the final analysis.

### 2.3. Survey Instrument and Data Collection

The online questionnaire was designed using Survey Monkey (Plus version, Palo Alto, CA, USA) and included 12 questions, structured into four groups: group 1—participant identification, stakeholder classification and sectoral involvement, group 2—importance of NIMCP, evaluation of NIMCP objectives (using a comparative ranking scale), group 3—perceived compliance with NIMCP objectives (assessed by a 5-point Likert scale), group 4—barriers and alternative policy measures, identification of key obstacles and potential solutions. A pilot test with 20 respondents was conducted prior to full deployment to ensure clarity and reliability. Pilot responses were not included in the final analysis.

### 2.4. Data Analysis

Quantitative data were analyzed using descriptive statistics and the Analytic Hierarchy Process (AHP), implemented through Expert Choice software (ver. 11.5, Arlington, VA, USA). The AHP method was used to compare stakeholder priorities and weigh policy objectives based on perceived importance. Responses were aggregated and analyzed to determine consensus and divergence among stakeholder groups. The Analytic Hierarchy Process (AHP) was used to quantify stakeholder priorities through pairwise comparisons of NIMCP objectives and improvement measures. Participants rated the relative importance of criteria using a 9-point scale [15]. Weights were derived from eigenvalue-based prioritization, with consistency ratios (CR) < 0.1 indicating acceptable judgment coherence. Aggregated weights reflect the geometric mean of priorities across stakeholder groups, normalized to sum to 1 for each hierarchy level. Sensitivity analysis confirmed the stability of the results.

The AHP weights were generated using Saaty’s eigenvector method. Stakeholders evaluated the relative importance of NIMCP objectives (e.g., “risk control” vs. “plant health”) on a 1–9 scale. Eigenvectors from the pairwise comparison matrices were normalized to produce priority weights (summing to 1 per hierarchy level), with a consistency ratio (CR) < 0.1 ensuring logical consistency.

To evaluate differences in stakeholder responses across categorical variables, chi-square tests were performed. Post hoc pairwise comparisons with Bonferroni-adjusted *p*-values identified specific differences between response categories. Analyses were conducted using JAMOVI R for Windows (version 19.0), with significance set at *α* = 0.05.

## 3. Results

### 3.1. Participant Demographics and Stakeholder Distribution

A total of 217 stakeholders participated in the study, providing a comprehensive representation of the food safety governance landscape in Portugal. The respondents (Table 1) were categorized into five stakeholder groups: (i) associations of animal production (comprising associations, federations, and cooperatives involved in livestock and agricultural production), (ii) private organizations related to animal production (including CEOs and companies engaged in animal husbandry and food production), (iii) public agencies related to animal production (including government institutions responsible for regulating and supporting food production activities), (iv) consumers and Consumers’ associations (including consumer rights organizations and individual consumers with an interest in food safety policies), (v) public food safety control agencies (including regulatory bodies such as inspection and enforcement agencies responsible for ensuring food safety compliance). This distribution ensured a diverse and balanced perspective on food safety policies across different sectors of the food supply chain. Although the study included stakeholders with varying levels of satisfaction with NIMCP implementation, participants were not explicitly classified according to their adverse relationships (e.g., tensions or rivalries) with regulatory agencies. Thus, studying the perceptions of stakeholders in active conflict with agencies and those aligned with them could be the subject of further research, which would contribute to elucidating tensions in food safety governance.

### 3.2. Stakeholders’ Perception About the NIMCP Objectives

Stakeholders were asked to assess the current level of compliance with NIMCP objectives using a 5-point Likert scale. Most of the responses were classified as “Mostly complied” and “Complied with as often as not,” indicating that respondents perceived a satisfactory level of compliance with NIMCP objectives (Table 2). However, the lower counts for “Always complied” suggest that full compliance has not been achieved. Regarding “Ensuring Animal Health,” 57 and 14 respondents indicated “Mostly complied” and “Always complied”, respectively, suggesting that stakeholders prioritize animal health measures. The lower response rate observed may be related to resource constraints faced by small-to-medium enterprises (SMEs) or the underrepresentation of plant-sector stakeholders, who accounted for only 21.7% of respondents.

In contrast, ensuring plant health and implementing official controls displayed the lowest compliance perception among respondents. The fact that Ensuring Plant Health received the highest number of responses for “Never complied” suggests that enforcing plant health control may be challenging. Additionally, Implementing Official Controls received a relatively high number of responses for “Rarely complied,” indicating difficulties in ensuring efficient and effective control implementation. The perception of consumer protection and food policy compliance among respondents showed moderate results, indicating room for improvement.

The analysis by chi-square test (χ^2^) revealed substantial disparities, with animal health objectives receiving significantly higher compliance ratings (53.8% combined “Mostly”/”Always” complied) compared to plant health objectives (39.4%, *p* < 0.01).

The Cronbach’s alpha for Stakeholders’ perception about the NIMCP objectives was 0.985, indicating excellent internal consistency. This suggests a strong correlation among responses, reflecting a consistent perception of institutional performance across the evaluated objectives.

### 3.3. Stakeholders’ Perceptions About Barriers to Effective Implementation of NMCPI

The most frequently reported barriers (Table 3) to the implementation of the NIMCP were “Lack of communication between public entities” (77 responses, 19.7%) and “Dispersion of responsible public bodies” (76 responses, 19.5%), indicating that coordination and communication among public institutions are significant challenges. “Insufficient information for operators” (51 responses, 13.1%) and “Limited communication with consumers” (47 responses, 12.1%) highlight deficiencies in the dissemination of information to key stakeholders. Additionally, “Lack of professional training” (42 responses, 10.8%), “Lack of action planning” (41 responses, 10.5%), “Technical incompetence of official services” (33 responses, 8.4%), and “Lack of human and material resources” (23 responses, 5.9%) were mentioned less frequently by respondents. However, all of these barriers relate to the government’s capacity to implement the NIMCP. As a result, approximately 25% of respondents perceive an inability of the state to effectively manage both human and material resources in the implementation of the NIMCP. Results are in accordance with the chi-square test that indicates systemic barriers like inter-agency communication gaps (19.7%) and institutional dispersion (19.5%) were reported significantly more frequently than resource limitations (5.9%, *p* < 0.001). 

The analysis of the main barriers identified by stakeholders resulted in a Cronbach’s alpha of 0.900, indicating high internal consistency among the items and suggesting that perceptions regarding the obstacles to the implementation of the NIMCP are largely aligned.

### 3.4. Stakeholders’ Perceptions About Alternatives to Improve the Implementation of NMCPI

Regarding the proposed alternatives for improving the NIMCP (Table 4), coordination and planning of actions (71 respondents, 22.2%) was the most favored alternative, suggesting that participants see value in structured and organized approaches. The emphasis on awareness campaigns for operators highlights the importance of educating and informing those involved in the process to ensure proper adherence and implementation. A significant number of participants suggested that creating an integrated network is crucial to connect different agencies, unify inspection processes, improve transparency, and enable the generation of rapid reports.

The development of a training plan (49 respondents, 15.3%) was also identified as essential; given that the lack of human resources has been identified as a barrier, the need for more skilled personnel is critical to ensure the proper implementation of the NIMCP. Periodic monitoring meetings (44 respondents, 13.8%) were seen as a valuable means to ensure that the plan stays on track. This suggests a need for regular follow-up to evaluate progress and make adjustments based on new food policies and/or technical knowledge.

The separation between risk assessment and risk management (24 respondents, 7.5%) was mentioned less frequently, but is aimed at improving focus and clarity in dealing with risks. Improving human and material resources (two respondents, 0.6%), although scarcely referenced, is associated with the previously described “development of a training plan” and the need for technical resources to implement the NIMCP effectively.

Finally, the regulation of food safety consultant businesses (two respondents, 0.6%) was proposed. While most ideas for improving the NIMCP focus on enhancing governmental human and material resources to aid food businesses, this measure aims to regulate private food safety consulting activities. The goal is to ensure that these services align with the requirements set by authorities under the NIMCP controls. Overall, structural solutions (coordination/planning: 22.2%; awareness campaigns: 21.9%) were prioritized over technical interventions (IT network: 18.1%) (*p* < 0.001) or regulatory changes (consultancy regulation: 0.6%) (*p* < 0.001).

The analysis of the alternative measures proposed by stakeholders resulted in a Cronbach’s alpha of 0.826, indicating good internal consistency among the items and suggesting a coherent pattern in the proposals aimed at improving the NIMCP.

### 3.5. Analytic Hierarchy Process for Stakeholders AHP

The analytic hierarchy process (AHP) results (Table 5) present a ranking of different priorities among the five stakeholders regarding the objectives of the NIMCP. The weight values assigned to each criterion indicate their relative importance in decision-making.

The AHP was employed to quantify stakeholder priorities through pairwise comparisons of NIMCP objectives and improvement measures.

Risk control is the top priority for stk1, stk2, stk3, and stk5. Although STK4 ranked it second, risk control remains a consistently dominant factor across respondents.

Consumer protection and food policy compliance are also high priorities for all stakeholders. Consumers’ protection ranks first for stk4 and remains within the top four across all scenarios. Similarly, food policy compliance ranks second for stk1 and stk5, highlighting its significance in decision-making.

Guaranteeing plant and animal health is of lower importance, as these objectives mostly rank fifth or sixth in all scenarios. In contrast, the implementation of official controls holds a mid-level priority, suggesting that while it is considered important, it is not as highly prioritized as risk control or food policy compliance.

The AHP results emphasize that risk control is the most critical factor across different contexts, followed by consumer protection and food policy compliance, guaranteeing plant and animal health appears to be the least prioritized aspect. This suggests that decision-makers primarily focus on risk mitigation and regulatory compliance to ensure food safety and public health.

The AHP regarding the alternatives (Table 6) to improve the implementation of NMCPI showed that coordination and planning of actions is the top priority, indicating a strong consensus that improving this measure is essential for enhancing the NMCPI. Information campaigns for food business operators were ranked second, suggesting that better communication and education for food business operators are necessary to improve compliance with food policy. The creation of an integrated IT network was not considered a critical measure for improving the NMCPI, although stakeholders acknowledge the importance of digital infrastructure in enhancing processes. Strengthening collaboration with consumer organizations holds moderate importance, suggesting that collaboration may contribute to better NMCPI implementation and objective achievement. Other measures, such as periodic monitoring meetings, training and formation, and separation between risk assessment and risk management, are not considered priorities by stakeholders for improving the NMCPI. The AHP results suggest that stakeholders overwhelmingly prioritize better coordination and planning, followed by informing food business operators and improving IT infrastructure. The ranking reflects a stakeholder preference for systemic improvements and efficient communication over regulatory restructuring.

## 4. Discussion

The present study provides a comprehensive understanding of stakeholder perceptions regarding food safety governance in Portugal, particularly concerning the National Integrated Multi-Annual Control Plan (NIMCP). The results highlight both the strengths and weaknesses of the current system, as well as the barriers and potential improvements that could enhance the effectiveness of food safety policies. The diverse representation of stakeholders offered a broad perspective on food control strategies applied in Portugal from different viewpoints. The fact that the stakeholder sample is more oriented toward animal production (72.8%) reflects Portugal’s economic and regulatory emphasis on this sector but may underrepresent phytosanitary perspectives. While this is consistent with the NIMCP-based prioritization of zoonotic risks, future studies should include a greater proportion of plant sector stakeholders to validate the findings and explore perceptions of emerging risks (e.g., pesticide residues, mycotoxins). Overall, stakeholders generally perceive a satisfactory level of compliance with the NIMCP objectives, particularly in areas such as animal health and risk control. The importance attributed to risk control and animal health may be associated with the fact that most food hazards originate from agents present in animal-based foods, either as zoonotic agents or as causes of foodborne infections [16,17]. Furthermore, increased access to information has heightened consumer awareness of food safety risks [18].

Additionally, the emphasis on risk control and animal health may be linked to emerging concerns related to food safety, such as chemical hazards (e.g., polychlorinated biphenyls, pesticides, nitrosamines, additives), antimicrobial resistance, or new food processing technologies [19,20,21,22]. Moreover, the importance stakeholders place on risk control and animal health aligns with the One Health approach, which recognizes the interconnectedness of human and animal health [23].

However, the lower levels of perceived compliance in areas such as plant health and official controls suggest that these areas require more attention [24]. The lower priority given to plant health may stem from a lack of consumer and public sector literacy regarding biological and chemical hazards [25]. In contrast, stakeholders involved in plant production prioritized plant health over animal health. This prioritization may be explained by their awareness of the hazards affecting human health and their perception that current controls are insufficient, leading to inadequate risk management, as described elsewhere [26].

The lack of prioritization of plant health by most stakeholders aligns with the perception that food safety measures for plant-based products do not meet NIMCP objectives, likely due to a lack of literacy in this area [27]. This perception could have serious implications for food safety and agricultural sustainability. Consequently, emerging plant hazards (e.g., viruses) may compromise food security, highlighting the challenges of managing plant health risks in the context of global trade [28].

The deprioritization of plant health despite low compliance reflects systemic biases in risk perception and governance. First, zoonotic risks from animal products dominate public health importance due to their direct relationship with foodborne diseases [1], while hazards related to plant-based foods (e.g., pesticide residues, plant pathogens) are often perceived as environmental or trade-related issues. Second, Portugal’s inspection system allocates fewer resources to phytosanitary controls, as evidenced by the dispersion of responsibilities across agencies. This mirrors trends at the EU level, where plant health receives between 12% and 18% of official control budgets, compared to 30% and 40% for animal health [26,29]. However, climate change and global trade are intensifying phytosanitary risks. Therefore, the undervaluation by stakeholders may be due to insufficient awareness of these emerging threats, highlighting the need for targeted risk communication, especially targeting consumer groups that gave plant health the lowest rating [27]. Thus, within the One Health concept, the inclusion of plant health would allow for greater emphasis on the impact of the hazards of plant-based foods on public health.

The challenges in implementing official controls, reflected in the high number of “Rarely complied” responses, point to systemic inefficiencies in control and inspection processes. Although difficult to explain, this could be attributed to several factors, such as a lack of technical knowledge, limited human and material resources in government agencies, or the impossibility of monitoring all food business operators, factors that align with the technical barriers described below [30,31,32]. Furthermore, insufficient knowledge about official food control schemes [24] or recent food scandals [33] may contribute to these perceptions.

Additionally, a lack of communication and/or transparency regarding how food hygiene inspections are conducted and the factors influencing inspection schedules may affect stakeholders’ perceptions of official control implementation [34]. These findings align with previous studies that have identified gaps in the implementation of food safety regulations, particularly in developing and middle-income countries [35]. The moderate perception of consumer protection and food policy compliance further underscores the need for more robust enforcement mechanisms and greater transparency in food safety governance.

Regarding barriers, “lack of communication between public entities” and “dispersion of responsible public bodies” were identified as major obstacles, suggesting that fragmented governance structures and poor inter-agency collaboration hinder the effective execution of food safety policies [36]. This is consistent with findings from other studies that have highlighted the importance of integrated and coordinated approaches in food safety governance [37]. Additionally, the European Food Safety Authority (EFSA) has emphasized the need for better coordination among member states to ensure the effective implementation of food safety regulations [38]. Furthermore, a unification of the inspection processes also allows for improved implementation of the NIMP through better management of human and material resources [30]. This dispersion of entities means that the scheduling of inspections is not homogeneous over time, limiting their effectiveness [39].

A comparative analysis reveals that NIMCP implementation challenges in Portugal reflect structural patterns observed in EU food safety systems. The German Kontrollverbund (a collaborative network overseeing official inspection and control of food, agriculture, feed, and consumer protection) presents analogous fragmentation problems [40], particularly in inter-institutional coordination, reflecting our respondents’ identification of the “dispersion of public bodies” as a key barrier (Table 3). In contrast, more integrated models, such as those observed in Spain [41] or France [42], show how centralized IT systems and clear risk assessment protocols can improve coordination between stakeholders, directly supporting Portuguese stakeholders’ proposals for digital integration.

Additionally, the lack of professional training and insufficient information for operators further exacerbate these challenges, indicating a need for capacity-building initiatives and better dissemination of information to key stakeholders. Thus, differences in opinion between inspectors can also mean inconsistent expectations and inconsistent focus on particular food safety issues, as well as related to lack of transparency of inspections and/or the behavior expressed by inspectors [43,44]. The relatively lower priority given to “Lack of human and material resources” suggests that while resource constraints are a concern, stakeholders view systemic issues such as communication and coordination as more pressing.

Among the improvement measures proposed by stakeholders for improving the NIMCP, they highlight the importance of more effective coordination, planning, and communication. The most indicated improvement measure, “coordination and action planning,” underscores the state’s need for a more structured and organized food safety control system. These improvement measures are related to the barriers mentioned above, reinforcing the consistency of perceptions among the various stakeholders participating in the study. The need for improved coordination may be related to the number of inspections carried out on economic operators, since these require time and, in many cases, money to implement the indicated reforms. Thus, a greater number of inspections carried out by different entities but with the same purpose makes the operator’s work more difficult, since many of these companies, mostly SMEs, do not have personnel dedicated to food safety. Furthermore, a greater number of inspections does not appear to improve food safety [45]. Some stakeholders, particularly from private sector groups, attributed compliance challenges to resource constraints disproportionately affecting SMEs, consistent with prior findings about inspection burdens on smaller operators [39,45]. Notably, 37% of surveyed producers reported that duplicative inspections from multiple agencies created operational burdens without perceptibly enhancing safety outcomes, a perception aligning with critical analyses of inspection efficacy in resource-constrained settings [34,39].

The improvement in communication reported by stakeholders also stems from this lack of coordination, where several regulatory bodies often have different interpretations [46,47] on the same point, which leads to greater efforts by companies to respond to official entities. Related to the lack of coordination, the lack of an interconnected network (i.e., database) prevents an organized overview of the food safety status of each economic operator, which diminishes transparency and trust not only between companies but also for consumers. Furthermore, a unified database would streamline inspection processes, facilitate the rapid presentation of results, and improve the management of human and material resources by the state [48].

The importance given to “Information campaigns for food business operators” and the “Creation of an integrated IT network” plan highlights the fundamental role of training in ensuring compliance with food policy, ensuring the food safety of processes and products, and avoiding problems and potential fines [49]. These improvement measures would also aim to overcome some of the difficulties identified by stakeholders (i.e., technical incompetence and insufficient information), ultimately leading to a more effective implementation of the NIMCP. The lower importance indicated by stakeholders regarding the “separation between risk assessment and management” may be related to the perception of sufficient information on food-borne hazards, as well as stakeholders’ perceptions of the capacity of official authorities to manage these hazards [50,51]. However, this lower importance may also be related to food safety literature [52,53] as well as to the social aspects of the respondents [54]. The AHP results further reinforce the importance of risk control and consumer protection as top priorities for all stakeholder groups. This is consistent with the broader literature on food safety governance, which emphasizes the need to mitigate public health risks and ensure consumer trust in the food system [31]. The lower priority given to plant and animal health, although somewhat surprising, may be related to a perception of relative safety given the scarcity of outbreaks and food recalls, as well as their media coverage [55,56,57]. Furthermore, this lower perception also suggests a possible lack of awareness of the interconnectedness of animal, plant, and human health within the One Health framework [58].

The AHP results regarding alternatives for improving the NIMCP indicate a strong consensus that better coordination and planning are essential to improving food safety governance. This is consistent with research that has highlighted the importance of integrated approaches to food safety management [59]. Also, interdisciplinary and trans-sectoral policy integration combined with increasing stakeholder collaboration across all sectors of the economy have been reported [14]. The emphasis on information campaigns for food business operators suggests that better communication and training are needed to improve food law compliance [60]. This is consistent with studies that have shown that effective communication strategies can improve stakeholder engagement and compliance with food safety regulations [46].

## 5. Conclusions

In conclusion, this study provides a comprehensive assessment of stakeholder perceptions regarding food safety control in Portugal. While the perception of compliance with the NIMCP objectives is positive, stakeholders also expressed concern about the limitations in its implementation associated with the lack of organization of state-run regulatory bodies and the lack of adequate communication regarding the different aspects of food safety. Thus, the improvement measures indicated by stakeholders include improvements in coordination, communication, and the development of activities such as awareness campaigns and training. However, some limitations may be considered. Thus, the stakeholder sample focused primarily on animal production sectors (72.8% of respondents), reflecting their dominant role in Portugal’s agri-food economy and the NIMCP’s emphasis on zoonotic risks. Although this design choice aligns with national priorities, future studies could broaden stakeholder representation from the plant sector to enable broader comparisons. The perception-based design elicits essential stakeholder views, with mixed-methods analysis (AHP + qualitative data) strengthening validity. Non-response rates (≤ 41% for optional questions) align with similar policy surveys and were addressed through sensitivity checks. These methodological choices enabled a rigorous analysis of the NIMCP’s core challenges, while identifying feasible improvements. The findings remain robust to Portugal’s key food safety priorities, and the stakeholder-driven framework serves as a model for other EU Member States. The measures proposed by stakeholders, particularly centralized coordination and integrated IT systems, provide viable policy tools to address documented shortcomings in NIMCP implementation. Thus, the results suggest reforms to NIMCP implementation and enforcement to optimize food governance in Portugal. By addressing these challenges, policymakers can strengthen the food safety system, thereby protecting public health and ensuring consumer confidence in the food supply chain.

## Figures and Tables

**Table 1:** Stakeholders’ characteristics.

Respondent’s Classification by Sector		N	%
	Associations of Animal Production	42	19
	Private Sector Animal Producers	60	28
	Public Sector Animal Production Agencies	31	14
	Consumers and Consumer Associations	58	27
	Public Food Safety Control Agencies	26	12
Respondent’s classification by position in the food chain			
	Primary production	82	37.8
	Transformation sector	27	12.4
	Distribution	19	8.8
	Retail	18	8.3
	Consumer	70	32.3
	Other ^1^	61	28.1
Respondent’s classification by origin of food products			
	Animal and animal-derived products	190	76.6
	Vegetables and vegetable-derived products	58	23.4
Respondent’s classification of food from animal origin			
	Meat and poultry	152	36.0
	Fisheries	74	17.6
	Dairy	87	20.6
	Eggs	59	14.0
	Honey	50	11.8

^1^: includes: livestock and farmers associations, private food laboratories, food certification and audit consultants, food safety training and education services.

**Table 2:** Stakeholders’ perceptions * about the NIMCP objectives.

	Never	Rarely	As Often As Not	Mostly	Always
Controlling Risks (Preventing, eliminating, or reducing risks to humans and animals to acceptable levels)	1 (0.8)	22 (16.7)	36 (27.3)	63 (47.7)	10 (7.6)
Promoting Legal Compliance (Ensuring that operators throughout the food chain comply with legal obligations)	1 (0.8)	17 (12.9)	49 (37.1)	58 (43.9)	7 (5.3)
Implementing Official Controls (Developing an efficient and effective control system through integrated planning and execution)	2 (1.5)	25 (18.9)	41 (31.1)	54 (40.9)	9 (6.8)
Defending Consumers (Ensuring fair practices in the trade of food and animal feed and protecting consumer interests)	2 (1.5)	23 (17.4)	56 (42.4)	41 (31.1)	10 (7.6)
Ensuring Plant Health (Ensuring official plant health control and preventing the entry of harmful organisms into the European Union)	5 (3.8)	23 (17.4)	52 (39.4)	45 (34.1)	7 (5.3)
Ensuring Animal Health (Ensuring animal protection and health, controlling animal diseases, zoonoses, and promoting animal welfare)	2 (1.5)	18 (13.7)	41 (31.1)	57 (43.2)	14 (10.6)

* Respondents’ responses: 132 (60.83%). Responses skipped: 85 (39.17%). NIMCP: National Integrated Multiannual Control Plan.

**Table 3:** Stakeholders’ perception (n, %) * about barriers in the implementation of the NIMCP.

Lack of Communication Between Public Entities	77 (19.7)
Dispersion of responsible public bodies	76 (19.5)
Insufficient information for operators	51 (13.1)
Limited communication with consumers	47 (12.1)
Lack of professional training	42 (10.8)
Lack of action planning	41 (10.5)
Technical incompetence of official services	33 (8.4)
Lack of human and material resources	23 (5.9)

* Respondents’ responses: 128 (58.99%). Responses skipped: 89 (41.01%). NIMCP: National Integrated Multiannual Control Plan.

**Table 4:** Stakeholders’ alternative measures proposed (n, %) * for improving NIMCP implementation (expressed as n, %).

Coordination and Planning of Actions	71 (22.2)
Awareness campaigns for operators	70 (21.9)
Creation of an integrated it network	58 (18.1)
Development of a training plan	49 (15.3)
Periodic monitoring meetings	44 (13.8)
Functional separation between risk assessment and risk management	24 (7.5)
Improve human and material resources	2 (0.6)
Regulation of food safety consultancy business	2 (0.6)

* Respondents’ responses: 128 (58.99%). Responses skipped: 89 (41.01%). NIMCP: National Integrated Multiannual Control Plan.

**Table 5:** Ranking * of stakeholders’ priorities of the NIMCP.

	STK1 Animal Production Associations	STK2 Livestock Farmers	STK3 Public Agents of Animal Production	STK4 Consumers and Cons. Assoc.	STK5 Public Agents of Official Control
Rank 1	Risk control (0.375)	Risk control (0.276)	Risk control (0.208)	Consumer protection (0.248)	Risk control (0.454)
Rank 2	Food policy compliance (0.229)	Guarantee plant health (0.158)	Guarantee plant health (0.163)	Risk control (0.161)	Food policy compliance (0.140)
Rank 3	Consumer protection (0.118)	Guarantee animal health (0.158)	Guarantee animal health (0.163)	Implementation of official controls (0.161)	Implementation of official controls (0.133)
Rank 4	Implementation of official controls (0.117)	Consumer protection (0.158)	Consumer protection (0.163)	Food policy compliance (0.161)	Consumer protection (0.126)
Rank 5	Guarantee plant health (0.081)	Implementation of official controls (0.129)	Implementation of official controls (0.163)	Guarantee plant health (0.134)	Guarantee plant health (0.078)
Rank 6	Guarantee animal health (0.080)	Food policy compliance (0.123)	Food policy compliance (0.140)	Guarantee animal health (0.134)	Guarantee animal health (0.069)

Same color indicates the same priority. * The weight values assigned to each criterion indicate their relative importance in decision-making. STK: stakeholders. Cons. Assoc.: consumers’ associations. NIMCP: National Integrated Multiannual Control Plan.

**Table 6:** Ranking * of stakeholders’ alternative measures (n,%) proposed to improve the NIMCP.

	STK1 Animal Production Associations	STK2 Livestock Farmers	STK3 Public Agents of Animal Production	STK4 Consumers and Cons. Assoc.	STK5 Public Agents of Official Control
Rank 1	Coordination and planning of actions (0.297)	Coordination and planning of actions (0.299)	Coordination and planning of actions (0.300)	Coordination and planning of actions (0.303)	Coordination and planning of actions (0.294)
Rank 2	Information campaigns for food business operators (0.236)	Information campaigns for food business operators (0.228)	Information campaigns for food business operators (0.227)	Information campaigns for food business operators (0.225)	Information campaigns for food business operators (0.239)
Rank 3	Creation of an integrated IT network (0.168)	Creation of an integrated IT network (0.174)	Creation of an integrated IT network (0.176)	Creation of an integrated IT network (0.177)	Creation of an integrated IT network (0.167)
Rank 4	Strengthened collaboration with consumer organizations (0.125)	Strengthened collaboration with consumer organizations (0.123)	Strengthened collaboration with consumer organizations (0.122)	Strengthened collaboration with consumer organizations (0.121)	Strengthened collaboration with consumer organizations (0.126)
Rank 5	Periodic monitoring meetings (0.091)	Periodic monitoring meetings (0.091)	Periodic monitoring meetings (0.091)	Periodic monitoring meetings (0.092)	Periodic monitoring meetings (0.091)
Rank 6	Training and formation (0.060)	Training and formation (0.060)	Training and formation (0.061)	Training and formation (0.061)	Training and formation (0.060)
Rank 7	Separation between risk assessment and risk management (0.024)	Separation between risk assessment and risk management (0.023)	Separation between risk assessment and risk management (0.023)	Separation between risk assessment and risk management (0.023)	Separation between risk assessment and risk management (0.023)

Same color indicates the same alternative measure. * The weight values assigned to each criterion indicate their relative importance in decision-making. STK: stakeholders. Cons. Assoc.: consumers’ associations. NIMCP: National Integrated Multiannual Control Plan.

## Data Availability

The original contributions presented in this study are included in the article. Further inquiries can be directed to the corresponding author.
